# Determination of unbound-bound moisture interface of faecal sludges from different on-site sanitation systems

**DOI:** 10.1016/j.heliyon.2025.e42091

**Published:** 2025-01-17

**Authors:** Arun Kumar Rayavellore Suryakumar, Edwina Mercer, Jonathan Pocock, Santiago Septien

**Affiliations:** a WASH R&D Centre, Dept. of Chemical Engineering, University of KwaZulu-Natal, Durban, South Africa; b Department of Chemical Engineering, University of KwaZulu-Natal, Durban, South Africa

**Keywords:** Batch settling test, Centrifugation, Differential scanning calorimetry, Faecal sludge, Gel point, Moisture boundness, Thermogravimetric analysis, Water activity

## Abstract

Non-sewered sanitation (NSS) has been adopted by a significant population, especially in developing countries. For the effective operation of NSS, it is important to ensure optimal treatment of the collected faecal sludge (FS) in on-site sanitation systems. Solid-liquid separation is among the most important steps in the treatment of FS in NSS. The ability of the FS for solid-liquid separation has assumed greater significance in the design and operation of sludge treatment process. Although dewatering indices such as specific resistance to filtration (SRF) and capillary suction time (CST) have been extensively used to determine the limits of sludge dewaterability, these parameters do not provide information on the limits of unbound moisture.

The unbound and bound moisture fractions of the FS samples were determined using different methods, and each method was evaluated for reliability. Different FS samples were collected from ventilated improved pit (VIP) latrines, urine diversion dehydrating toilets (UDDT) and septic tanks (ST). Batch settling tests (BST), centrifugation, thermogravimetric analysis (TGA), differential scanning calorimetry (DSC) and water activity (WA) measurements were performed on the samples. While the BST provided the settleability of FS at gravitational force, and centrifugation estimated the relative limit of solid-liquid separability upon application of a force, the boundary of unbound moisture or the interface of unbound and bound moisture fractions as determined by TGA, DSC and WA. Evidently there was a fine overlap of the unbound and bound moisture at the interface due to presence of some tightly held unbound moisture and loosely held bound moisture. The unbound-bound moisture interface, thus, can be experimentally determined to be between 51.07 and 60.35 % for VIP sludge, 51.48–64.38 % for UDDT sludge, 62.29–66.71 % for ST-wGW sludge and 59.11–60.37 % for ST-GW sludge. Although hypothesis testing revealed no statistically significant difference between the methods and sample types, it can be concluded that WA demonstrated the highest reliability in terms of accuracy, ease of measurement, rapidity and repeatability.

## 1 Introduction

Sustainable Development Goal 6 (SDG 6) emphasizes universal access to safe sanitation with access to toilet as the first step to ensure no open defecation occurs, and goes beyond addressing the broader challenge of providing access to safe management of the faecal matter [1]. At places where the centralized sewerage network is either absent, un-implementable or un-affordable, the non-sewered sanitation system has emerged as a preferred sustainable option, with on-site sanitation systems (OSS) become the pre-dominant form of storage of faecal sludge (FS) at the point of generation. Over 56 % of the world's population in about 58 countries (1.8 billion people) have reported the use of such OSS facilities. Ventilated improved pit (VIP) latrines, urine diverting dehydrating toilets (UDDT) and septic tanks (ST) are amongst the common OSS. The FS can be raw or partially digested, can be in the semisolid or slurry form, with or without urine, and with or without greywater [2]. A comprehensive faecal management system (FSM), includes safe containment, reliable transport, effective treatment and safe end-use or disposal [3]. OSS forms part of the sanitation value chain, which further includes a safe conveyance infrastructure and a treatment facility, when they eventually get filled up [4].

The moisture content of FS varies between 99 % and 65 % depending upon various reasons, mainly on the type of the containment system and the practices of the population with regard to the use of water [5,6]. Due to the high moisture content, the solid-liquid separation process becomes one of the critical steps in the treatment of FS. Removal of moisture from the sludge helps to lower energy demands for further treatment; helps volume reduction; reduces treatment footprint and encourages circularity by better resource recovery. Further, the solid and liquid fractions after separation, can be effectively treated [4,7]. The different types of solid-liquid separation process for sludges can be broadly classified into different types based on the working principle of the process: a) settling and thickening (gravitational dewatering); b) mechanical dewatering; and c) thermal drying. While the most common are settling-thickening tanks, and sludge drying beds, because of its simplicity and low energy costs, mechanical dewatering and thermal drying are more energy intensive, but also can remove more moisture from the sludge.

The moisture in sludges is classified into different types based on the binding interaction with the solid particles. The types of moisture, thereby considered in this research, are as follows [[8], [9], [10]].

1. Unbound moisture or free moisture
2. Bound moisture, which includes sub-fractions of interstitial moisture, vicinal moisture and intracellular moisture.

While the unbound moisture is free and devoid of any binding energy, the bound moisture exhibits distinct binding energies for the different sub-fractions and the binding energy tends to increase with increasing solids concentration. The presence of binding nature of the moisture is of particular significance due to the operational challenges of the sludge resulting in stickiness, flowability and the increase in the carbon footprint due to higher energy costs for treatment [11]. With the moisture binding energy at zero, it is said that the unbound moisture can be removed from the sludge relatively easily than the bound moisture with positive moisture binding energy. Understanding the unbound-bound moisture interface within the sludge is, thus, central for solid-liquid separability process. The multi-analytical methods to verify the efficacy has been commonly used by researchers for environmental applications [12]. The early tests used to determine the moisture fractions in sewage sludge were the drying tests [8], differential scanning calorimetry [13] and water activity [14]. The relevance of these instrumentation approaches for FS was assessed in this research. While the moisture content of sewage sludge varied between 78 % and 99.5 %, the bound moisture fraction was typically around 30 % of the total moisture content of the raw sludge [15]. The moisture fractions of sewage sludges are different to that of FS because of the high variability of FS characteristics.

The determination of unbound and bound moisture fractions thus forms the scope of this research. The research encompasses FS from different OSS collected from South Africa (ventilated pit latrines, urine diverting and dehydrating toilets and septic tanks), which are prevalent in the non-sewered sanitation nations. While the batch settling tests and centrifugation tests provide the fundamental basis for solid-liquid separation under gravity and centrifugal force respectively, the TGA, WA and DSC go beyond the limits of dewaterability, to provide the unbound-bound interface of the sludge. The huge variability of FS and the intrinsic principle of the experimental methods form the limitations. With the overall research goal to determine all the moisture fractions of FS, the objective of this study, was focused to determine the proportions of unbound and bound moisture fractions in the different FS samples by different approaches and evaluate the relative pros and cons of the determining tests. Ethical clearance from the Biomedical Research Ethics Administration (BREC) from the University of KwaZulu-Natal, was obtained for this research (protocol reference number: BREC/00002194/2020).

## 2 Materials and methods

### 2.1 Sample collection and preparation

The faecal sludge types used as feedstock for this study were selected to represent the non-sewered sanitation systems in sub-Saharan Africa and South Asia.

1. Faecal Sludge from ventilated pit latrines (VIPs)
2. Faecal Sludge from urine diversion dehydrating toilets (UDDTs)
3. Faecal Sludge from septic tanks (also referred as septage)
  a With grey water (ST-wGW)
  b Only black water (ST-BW)

The samples were collected from the peri-urban areas, under the administrative jurisdiction of eThekwini Municipality, Durban and from Pietermaritzburg, South Africa. The samples were collected in lined plastic containers, with air-tight lids, screened for trash and debris, and stored in cold room at the laboratory at 4^o^C in order to minimize microbial degradation and avoid loss in moisture, if any.

The VIP latrines were desludged more than 5 years ago. The sample were collected from two pits, from the middle which should represent a sludge age of about 2–2.5 years. The pits were emptied manually, using shovels. In previous studies, it was observed that there has not been considerable difference in the characteristics of the sludge across the front side and the backside of the pit, and with regard to the depth, except for slightly lower moisture content = at the bottom sludge fraction [16]. Though the sludge did have large solid waste debris, care was exercised to avoid them in the samples.

The UDDT featured a waterless system with two vaults – active vault which collects the faeces, and once full, the pedestal is moved over to the adjacent vault. While the second vault is in use, the content of the first vault is shut for dehydration. When the second vault is full, which is designed to take 10–12 months, the dehydrated contents from the first vault are removed, and the pedestal is moved back on the first vault. The samples were collected from the dehydrating vault of two toilets, with an estimated storage time of about 12–18 months. The users of the toilet have not used any dehydrating material (like saw dust, ash, etc.). The cover to the vault was broken and some solid wastes like plastics and others were found in the sludge [17].

Septic tanks are another type of OSS, which are classified as passive low-rate anaerobic digestor. They are usually two-chambered tanks, and designed to perform as a settling tank for solids present in the wastewater flow. The septic tanks are connected to either only to the toilet (blackwater) or to both blackwater and greywater – wash water, laundry, kitchen, etc. The design and sizing varies accordingly, due to change in the influent volume and characteristics of wastewater [18]. Samples from both types of septic tanks were collected – one with both blackwater and greywater flow, which is representative to the systems in Southern Africa, and another where only the toilets were connected to the septic tanks, representative of most septic tanks used in South-Asia.

Septic tanks are desludged by vacuum tanker trucks once every 3–5 years. Two septic tank samples were collected from the vacuum trucks, and for samples from septic tanks, a composite sample was prepared by collecting the septage at four different levels of flow when the collected sludge was discharged from the tanker at the treatment works. The composite sample consisted of a) sample at the beginning of the discharge; b) sample at 25 % of the discharge; c) sample at 50 % of the discharge; and d) sample at the end of the discharge [19].

The FS samples were tested for total solids (TS), volatile solids (VS), pH and electrical conductivity (EC) in accordance with the procedures defined in methods for faecal sludge analysis [20] and results are provided in Table 1. Each sample was tested in triplicate, and the average result was reported. The time interval of about 5 years between desludging allows natural degradation and consolidation to occur in VIP latrines, which was evident from the TS and VS/TS values. Though the UDDT and ST samples were not as old as VIP, the extent of anaerobic degradation and thereby, the stabilization of the sludges was evident from the results [18,21]. The moisture contents of the FS from different OSS corroborates with the FS characterization in different studies on VIP samples [22] and septic tank samples [23,24].

**Table 1:** Preliminary characteristics of FS samples.

Sl.	Sample	TS	MC	VS	VS/TS	pH	EC
		g/g	%	g/gTS	%	–	mS/cm
**1**	VIP	0.24	73.14	10.62	43.52	8.61	6.57
**2**	UDDT	0.25	68.85	10.66	42.96	8.81	13.84
**3**	ST - wGW	0.01	99.12	0.37	33.41	7.25	2.15
**4**	ST - BW	0.07	94.04	0.033	52.67	7.29	7.89

### 2.2 Experimental methods

#### 2.2.1 Batch settling tests

The organic sludges are known to the formation of a networked gel-like structure at lower solids' concentrations. Termed as gel point, the solid's concentration in the sludge start to form a networked sludge and define the point of resistance to any applied pressure. Determination of gel point for different FS was achieved from the gravitational batch settling tests (BST) to determine the settleable behavioural after undergoing consolidation in the OSS during the storage period [25]. For sewage sludge from an aerobic treatment facility, the typical solid's concentration when the networked layer forms was low, at around 1.5–4.0 wt% [26].

The gel point ϕ_g_ was determined from the final equilibrium settling height measured from a 500 ml cylinders, and is given by the equation:

(1) $$\emptyset_{g}\approx \emptyset_{\mathrm{av}}=\frac{\emptyset_{0}h_{0}}{h_{\infty }}$$

where $\emptyset_{0}$ and $h_{0}$ were the initial solids' volume fraction (%) and height (cm) respectively, while $h_{\infty }$ was the final equilibrium height (cm).

BST was performed for all the FS samples in triplicates until no change in the height of the solid-liquid interface was observed. Diluting the samples with their own supernatant liquor was necessary to calculate the particle interactions at the different solids’ concentrations. Since the FS samples had high TS, extraction of required quantities of liquor for dilution were not practically viable. As a substitute, a saline solution was used with same electrical conductivity (EC) as that of the liquor as verified and established by Ref. [27] for sewage sludges. This dilution prevented the cell lysis from osmotic pressure. The samples were first tested for EC to prepare the equivalent NaCl solutions for dilution. Approximately 50 g of the sample was mixed with the corresponding salt solution of same EC up to 500 ml. The samples were transferred to 500 ml measuring cylinders and sealed with parafilm. The matrix was mixed again by turning upside-down for re-suspension, and the decrease in the height of the solid-liquid interface was measured over time, extending into 4–5 weeks. The test was carried out in triplicates for all FS samples.

#### 2.2.2 Centrifugation

The centrifugation test was used to measure the extent of moisture removed by application of an external force, thus assessing the physical limit of dewaterability of the sludge. This is not the same as determining the unbound-bound moisture interface since most of the dewaterability techniques do not necessarily remove all of the unbound moisture. It has been observed that the dewatering process of sewage sludge using centrifugation achieve about 20–30 % of solid's concentration, leaving a viable gap of 10–40 % of moisture which can be removed but could take a long time [28]. Centrifugation is a conventional mechanical dewatering method, which removes the moisture from the wastewater sludges, consequently increasing the solid content in the sludge cake [29]. The experiment is based on the principle that as the centrifugal forces is increased, the free moisture gets withdrawn, and the sludge particles get more compacted under the influence of the centrifugal force. The extent of moisture removed was determined by measuring the cake's moisture content after centrifuging the samples at different revolutions per minute (rpm), and for different time durations. Upon centrifugation, the solid particles settle quickly since the gravitational acceleration, as in the case of BST, is much less than the centrifugation acceleration, and with the increasing centrifugation acceleration, the sludge continued to release moisture. However, for all practical purposes, there is a limit to increasing centrifugal acceleration [30].

About 28–30 g of VIP and UD samples, and 30 ml of ST sample were centrifuged in triplicates at 3000, 4000 and 5000 rpm for time intervals, from 20 to 100 min with 20 min intervals, using a table-top centrifuge *Hermle Labor Technik GmbH* (Wehingen, Germany), and the supernatant was removed and the moisture content of the cake was determined according to standard methods for FS analysis [20].

The relative centrifugal force (RCF) or g-force is the function of the speed of rotation in rpm, and is given by the equation:

(2) $$RCF={\left(RPM\right)}^{2}\times 1.118\times 10^{-5}\times r$$

Where r (cm) is the distance of the suspended sludge from the centre of the centrifuge and RPM is the number of rotations per minute. Correspondingly, the g-force values are 704×*g* for 3000 rpm, 1252×*g* for 4000 rpm and 1956×*g* for 5000 rpm.

#### 2.2.3 Thermo-gravimetric analysis

Thermo-gravimetric analyzer (TGA) is one of the most preferred techniques to explore drying kinetics, as it measures the mass change and its rate as a function of temperature, time and atmosphere. The mass change occurs by: decomposition – breaking of chemical bonds; evaporation – loss of volatiles with elevated temperature; reduction – interaction of the sample to a reducing atmosphere; and by adsorption or desorption. TGA can provide data to analyze thermal stability, oxidative stability, decomposition kinetics as well as moisture and volatile content volatilization of materials. It is capable of controlled and ramping temperatures and hold isothermally for fixed periods of time. In this study, the tests were employed to determine the loss of moisture of the sludge by evaporation, as a function of time [31,32].

The mass change analysis by evaporation was carried out *in Differential Thermal Analysis - Thermogravimetric Analyser SDT Q600* instrument (TA Instruments, USA) (DTA-TGA) at the Thermal Analysis Laboratory, Department of Mechanical Engineering, Durban University of Technology, Durban, South Africa. There were two balance beams in the *SDT Q600* instrument, where one acted as the reference while the other was used for the sample. The procedure used involved the placement of ceramic pans on each balance beam to tare. Using a spatula, the sample was then placed in one of the pans. A tweezer was used to place the pan on the beam balance. The samples were scanned using pure N_2_ as the carrier gas at a flow rate of 10 mL/min, with 35–40 mg of sample, and the cell temperature from ambient (25^o^C) to 90^o^C at a heating rate of 10 ^o^C/min and maintained in isothermal condition for 45 min. All samples were analyzed in duplicates.

The vapour pressure difference between the sludge and the ambient air is the driving force of drying, and the rate of change of the moisture rate characterizes the drying kinetics [6,33]. Therein, moisture ratio (MR) was used to compare the change in the loss of moisture over time, and calculated according to the equation:

(3) $$MR=\frac{M_{t}-M_{e}}{M_{o}-M_{e}}$$

Where MR is the moisture ratio, M_t_, M_o_ and M_e_ are the moisture content at time t (min), initial moisture content and equilibrium moisture content (all in %) respectively. Equation (3) is simplified to equation (4) since the N_2_ gas atmosphere is devoid of any humidity, and the sample can then be dried completely, implying an equilibrium moisture content equal to 0 [34,35].

(4) $$MR=\frac{M_{t}}{M_{o}}$$

The drying rate of the samples was calculated by the equation:

(5) $$DR=\frac{M_{t+dt}-M_{t}}{dt}$$

where M_t + dt_ (%) is the moisture content at time t + dt (min).

MR was plotted against the DR to measure the change in the drying rate as MR decreases over time.

Further, the relationship between MR and time was analyzed in Excel using the 2nd order polynomial regression, as expressed in Equation (7). The x value represents time (in min) and y represented the moisture ratio, and for the P1, P2 and P3 values, confidence interval of 95 % was specified. The polynomial regression helped define and estimate the selectivity co-efficients, with 95 % interval for the data [36,37].

(6) $$Linear\;Model\;Poly2\;y=P1x^{2}+P2x+P3$$

#### 2.2.4 Water activity

The water activity measurement was used to measure the thermodynamic activity of water versus the moisture content within the sludge, at a given temperature. Water activity is commonly used to correlate food safety and quality in the food industry. It is the measure of the state of moisture in the organic matter, and controlling water activity in foods is one of the preferred preservation methods to ensure food safety against microbial and chemical deterioration. For instance, very dry food with water activity <0.2 could be degraded by lipid oxidation, and the tissue foods such as red meat with water activity >0.98 would be spoilt by bacterial growth [38].

Water activity (WA) is defined as the ratio between the partial vapour pressure of moisture in the substance and the standard partial vapour pressure of pure water [39,40]. It is a function of moisture content and a good indicator of moisture boundness in the sludge, with high water activity close to 1 indicating presence of free or unbound moisture, and lower water activity measurement corresponding to lower moisture and strong boundness [6,9].

The FS samples were dried in an oven at 105^o^C, and at selected pre-defined time intervals during the drying process, well-mixed sub-samples were taken. The moisture content of the sub-samples was measured using a *Radwag Max 50 Thermal Moisture Analyser* instrument (RADWAG, Poland) before measuring the WA. The WA of these sub-samples with different moisture content was measured with a *AquaLab Tunable Diode Laser (TDL*) *water activity meter* (Meter Group, Pullman, WA, USA). This instrument adopts a spectroscopic approach to determine water activity, by measuring the absorption at 1854 nm of wavelength, which is specific to water vapour in the headspace of the sample. From this value, the relative humidity in the headspace can be determined. Since it is assumed that the sample is in thermodynamic equilibrium with the gas phase above it, the water activity can be assumed to be equal to the relative humidity in the headspace. The major advantage of TDL instrument is the direct measurement of vapour in the headspace independent of other volatile substances [41].

The temperature was set at 22^o^C with a resolution of ±0.0001 and accuracy of ±0.005. The range of the water activity measurement was from 1 to 0, and by plotting the water activity as a function of moisture content, the moisture vapour sorption curve of the FS samples was obtained. The temperature stability of the instrument and the instrument calibration was critical for TDL approach, and necessary precautions were taken, and repeated calibrations were made to ensure accuracy of the measurements. All samples were measured in triplicates.

#### 2.2.5 Differential scanning calorimetry

Unbound moisture in sludge freezes like normal water whereas bound moisture does not, and this behaviour of unbound moisture was measured using differential scanning calorimetry (DSC). DSC is a thermal analytical instrument that records the energy changes of the sample and determines the enthalpy of materials as a function of temperature. This instrument is used to measure physicochemical states such as the product stability, glass transition, melting and crystallization in chemical and process engineering. DSC is widely used in the biopharmaceutical industry, with proteins and peptides representing the largest class of materials analyzed. The temperature of the chamber can be constant or freezing/thawing at a specific temperature increase or decrease rate. DSC thermogram analysis were carried out in aluminum pans, with the instrument calibrated for temperature and heat flow both exothermically when the sample freezes and endothermically when the sample melts. This helps in the quantification of the amount of unbound moisture with the underlying assumption that the unbound moisture behaves as pure moisture and freezes and melts at temperatures close to 0^o^C [42]. Subtracting the unbound moisture from the total moisture of the sample provides the amount of bound moisture.

About 8–10 g of the sample was placed in the aluminum sample holder, having a central 0.7 mm pin hole. The DSC tests were conducted on the FS samples to observe the exothermic and endothermic peaks when the temperature of the sample was frozen up to −60^o^C. The thermograms were recorded between ambient temperature (25^o^C) to −60^o^C and −60^o^C to ambient at a heating/cooling rates of 10 ^o^C/min in a pure N_2_ atmosphere [13,32]. The experiments were conducted in a *DSC 600* instrument (PerkinElmer, USA) in duplicates.

The area under the endothermic and exothermic peaks in the DSC thermograms are indicative of the total heat absorbed and released, respectively. The measurement of this energy absorbed helps to quantify the amount of unbound moisture. The chemical potential of the bound moisture influences the freezing temperature of bound moisture in the DSC experiments. The resistance to freezing of the bound moisture at low temperature was attributed to its reduced chemical potential in the solid particle-moisture interactions, and this was measured in this study [13]. The trapezoidal rule was used to calculate the area under the curve to compute the specific enthalpy of reaction. The trapezoidal rule is a numerical method to have an approximation of an integral function and helps to integrate the area under the curve. This involved dividing the area under the curve into a number of trapezoids of equal width. The area of each trapezoid was computed, and the summation of all the areas provide the final numeric value [43] from Equation (7).

(7) $$T=\left(\sum_{i=1}^{n}\left(\frac{T_{i}+T_{i+1}}{dt}\right)\right)\times \left(\frac{1}{b-a}\right)$$

Where T is the freezing enthalpy of moisture (J/g), T_i_ and T_i+1_ are the enthalpies recorded at time t and next interval t+1 (min), a and b are the limits of the integration, and n is the number of intervals. The unbound moisture was calculated as a ratio of the enthalpy of heat of sample to the enthalpy of heat of pure water [44] and the bound moisture is given by Equation (8).

(8) Wb=1−(QendoQpure)

Where Wb is the bound water fraction (%); Q_endo_ is the enthalpy of heat of samples (J/g), from the DSC thermograms and Q_pure_ is the enthalpy of pure water, assumed as 334.6J/g.

### 2.3 Multi-variate statistical analysis

To test if the test methods could affect the unbound-bound moisture interface, a multi-factor analysis of variance (ANOVA) was used to test for the statistical differences between mean moisture content between the samples of unbound-bound moisture interface. The type 1 error rate was set at 0.05 for all the statistical tests performed in this study and was carried out using R software. The null hypothesis *H*_*0*_ was taken as no significant difference between results from different methods and between different sample types, and the alternate hypothesis *H*_*a*_ as having significant difference.

Test for normality (the samples are from population(s) that are normally distributed) and test for homogeneity (the variances are equal) are pre-requisites of ANOVA test. Shapiro-Wilk test, which was originally for data sizes less than 50, is used to check the normality of the data sets. Levene Test was used to check for homogeneity. After satisfactory outcome from the normality and homogeneity of variances tests, ANOVA was selected for statistical analysis to determine where there are any significant differences [45].

## 3 Results and discussion

### 3.1 Batch settling test

**Fig. 1:**
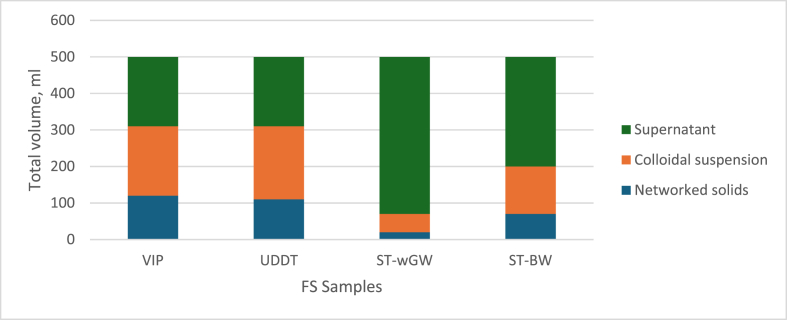
Batch Settling Test results for FS samples.

An upgraded version of sludge volume index experiment, the BST experiments determined the gel point thus defining the point of resistance of the sludge to any external and applied pressure besides gravitational force. The gel points of the FS samples represented the point of networked settleable behaviour of the samples under gravity force. The gel points for all the FS samples were clear with the settling in the cylinder exhibiting 3 distinct phases, which were – clear supernatant, networked solids at the bottom, and in-between these two, a layer of colloidal suspension (Fig. 1). The three phases were clear and visible prominently with both the septic tank samples. This could possibly be because of low initial total solids. It was visible on keen observation with the VIP and UDDT samples. This colloidal suspension which was prominent between the solids and supernatant with all the FS samples has not been reported with other sludges, and more exploratory research is required to understand this colloidal suspension and its possible relation to moisture boundness.

It can be noted that the gel points of the FS samples were at about half their respective initial TS, except for the ST-wGW sample which was at its TS level. The gel point of VIP sample was at 13.15%TS, UDDT sample at 14.06%TS, ST-wGW at 1.79%TS and ST-BW at 3.52%TS. Further, the gel point points for fresh faeces ranged between 6.3 and 15.6 % TS [46] similar to the VIP, UDDT and ST-BW samples. The ST-wGW sample exhibited relatively low gel point due to lower TS, and presence of greywater, and was similar to the gel point of sewage sludges (1.5–4.0%TS). Further, it was also assumed that the different retention times and the anaerobicity in the OSS have little or no influence on the gel point.

### 3.2 Centrifugation

Centrifugation increase the magnitude of the gravitational force, so that the particles in suspension experience the g-force. The centrifugation tests were carried out to determine the extent of moisture removal possible from the different FS by application of external force, since this was one of the widely available instruments in any environmental laboratory. There are two groups of moisture possible in a centrifuge, operationally speaking – one which was “mechanically removable moisture” and the other remaining in the sludge solids, as “trapped moisture”. While the “mechanically removable moisture” comprises only unbound moisture, the “trapped moisture” comprises all of the bound moisture and some unbound moisture. Centrifugation, thus, provides data on the extent of moisture removal possible from the sludges because the centrifugal acceleration which is higher than the gravitational acceleration [47].

**Fig. 2:**
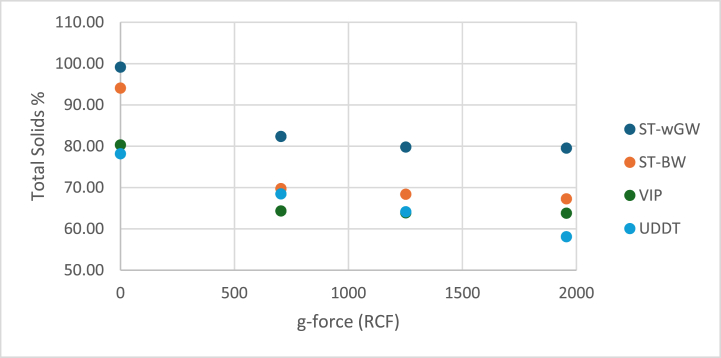
Centrifugation test of FS at different g-forces.

The centrifugation g-force (RCF) of 704×*g*, 1252×*g* and 1956×*g* for 100 min was plotted against the remaining moisture content in the solid pellet. The decreasing moisture content of the sludge with increasing centrifugation (Fig. 2) evidently exhibit the dependency of the moisture content on the centrifugal force. This decrease in moisture content continued as the centrifugal speed increased, up to with all unbound moisture was removed, along with perhaps some loosely bound interstitial moisture. After a certain point when the centrifugal force was increased further, the moisture removed reduced substantially, and the relationship of the moisture content to the centrifugal force went towards linear almost attaining a plateau beyond this point of rotational speed and time of centrifugation. This suggests that all “mechanically removable moisture” was mostly removed, and the remaining moisture was mechanically challenging to remove “trapped moisture”. For all the samples, after 60 min of centrifugation, the incremental moisture removed was less than 1.5 % for 80 min and 100 min. It can be said that at infinite time, all unbound moisture might be removed completely. However, for all practical purposes, this is not possible as the incremental increase in the moisture removed became less as time increased.

The moisture content of the cake obtained at 1956×*g* for 100 min for VIP sludge was 63.77 %, for UDDT sludge was 58.09 %, ST-BW sludge was 67.26 % and for ST-wGW sludge was 79.54 %. The VIP and UDDT samples, which had a higher initial TS, showed higher final TS in the pellet. The percentage of moisture removed from the samples was between 19 % and 28 %, with VIP sludge at 20.57 %, UDDT sludge at 25.68 %, ST-BW at 28.48 % and ST-wGW at 19.76 %. It can be concluded that the moisture removal cannot be predicted based on the type of OSS. Comparing with sewage sludge, the moisture removal for unconditioned aerobically digested sewage sludge, was at 15 %, still lower than the ST-wGW and VIP samples, whereas for dewatered anaerobically digested sewage sludge, the removal efficiency was around 30 %, similar to the UDDT and ST-BW samples [48].

### 3.3 TGA measurements

The variation of the moisture with drying time is known as the drying curve, which undergoes different phases. The graphical representation of rate of evaporation or moisture removal (drying rate) versus moisture content is given by Krischer's curve, which helps to detect the kinetic phase changes. The typical drying curve can be divided into constant drying rate period, first falling rate period, second falling rate period and equilibrium stage [49,50].

**Fig. 3:**
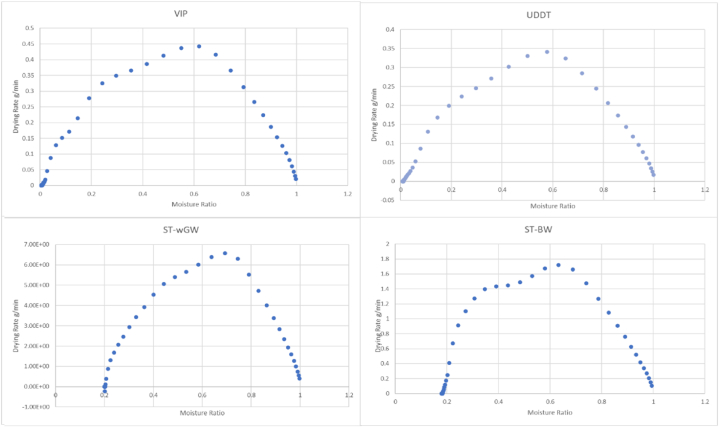
MR Vs DR curves for the different FS samples during the TGA tests.

The moisture ratio was plotted against mass loss to the initial mass per unit time (drying rate) with increasing temperature (Fig. 3). The results from the non-isothermal drying kinetics showed decrease in the sample mass due to moisture evaporation due to drying and finally tending to zero, as the drying got completed. Moisture reduction is accompanied by heat transfer, and the total mass loss to the sample agreed to its moisture content, as seen in Fig. 6. The drying rate increased initially during the warm-up period as the sample got heated up, and subsequently, the drying rate had both first falling rate period and second falling rate period, but without a constant drying rate period. The ramp-up period masked the constant drying rate period with increasing drying rate proportional to the increasing temperature, and the evaporation of the unbound moisture during this ramp-up [51,52].

The heat transferred from the gas phase to the inside of the sample by both conduction and convection, with the drying occurring initially at the outer skin of the sample, and then in the inner part. The inner moisture, hence, diffused mostly through the pores of the dried solid layer. This is similar to the drying mechanism of the unreacted core model as a gas-solid reaction model considering the zero order on gas reactant concentration and a solid particle of fixed size. This was evident from the falling drying rate periods from the drying curves even when the temperature was increasing. This was also observed by Ref. [53] for sewage sludge.

**Fig. 4:**
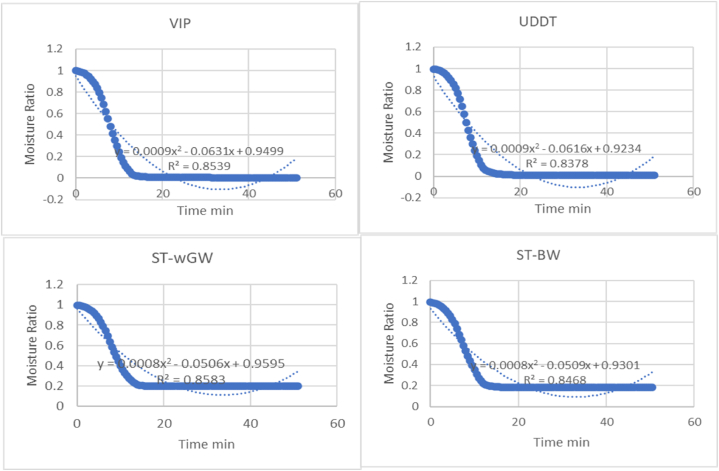
Moisture ratio vs time for the different FS samples.

From the polynomial analysis of MR versus time at 95 % confidence (Fig. 4), the R-squared values were higher than 0.85 for VIP and ST-wGW sludges, and at 0.83 and 0.84 for UDDT and ST-BW samples. The selectivity co-efficients and R-squared values indicate the data fitted well, as seen in Table 2, given the high variability of the FS.

**Table 2:** Selectivity co-efficients and R-squared values for MR vs time.

	VIP	UDDT	ST-wGW	ST-BW
P1	0.0009	0.0009	0.0008	0.0008
P2	0.0631	0.0616	0.0506	0.0509
P3	0.9499	0.9234	0.9595	0.9301
R-squared	0.8539	0.8378	0.8583	0.8468

### 3.4 Water activity measurements

At a given relative humidity and a defined temperature, it was possible to measure the water activity of the sample in its thermodynamic equilibrium with its atmosphere. The moisture sorption isotherm describes the thermodynamic activity of water versus the moisture content within the sludge, at a given temperature. The water activity measurements were close to 1 up to almost 55–60 % of moisture content of the FS samples where the partial vapour pressure of moisture in the sample was nearly equal to the standard vapour pressure of pure water. This was possible when there was free moisture in the sludge, without any binding energies. This corresponds to the constant drying rate phase in the conventional drying curve, matching to the presence of unbound moisture.

**Fig. 5:**
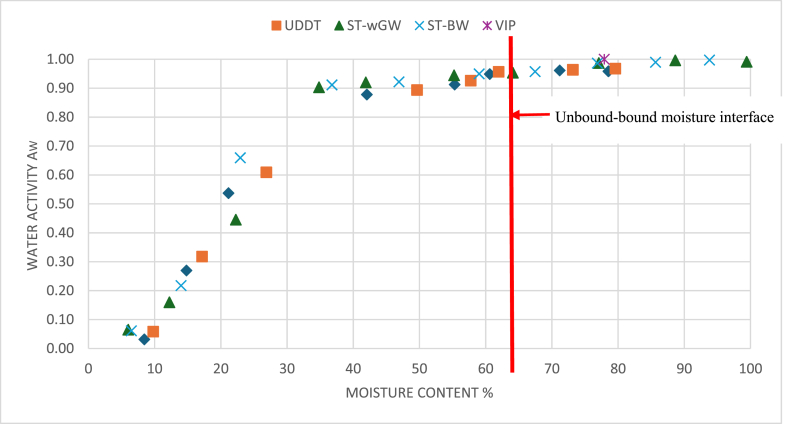
Water activity vs moisture content for the FS samples.

As the moisture content of the sample reduced, a gradual fall in the water activity measurements was observed (Fig. 5). This reduced vapour pressure of the moisture in the sludge corresponded to the first falling rate period of the drying test. There was no significant difference in the water activity measurements at different moisture content between the FS samples from different OSS [54].

**Fig. 6:**
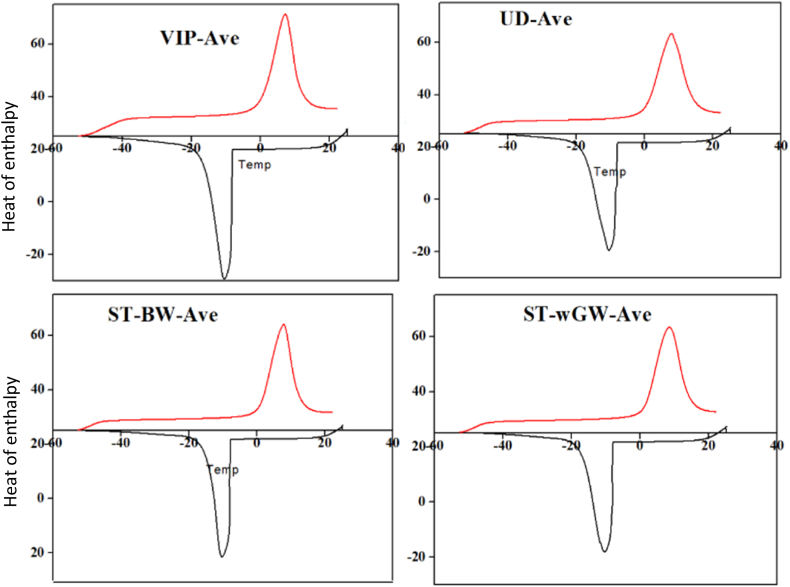
DSC thermograms for the FS samples.

### 3.5 DSC measurements

With the hypothesis that bound moisture does not freeze down to a threshold temperature, the application of DSC to determine the bound moisture content of FS samples provided a thermodynamic interpretation of unbound-bound moisture interface. The enthalpy was plotted against the freezing and thawing temperature (Fig. 6). The release of heat in the DSC thermogram indicated the presence of unbound moisture fraction and was equal to the latent heat required for freezing unbound moisture. Unbound moisture was thus easily differentiated in the DSC from the un-freezable bound moisture that did not freeze at normal freezing temperature of pure water. From the plot, as the temperature decreased towards −60^o^C, heat was released around -8^o^C to −20^o^C for all the FS samples. After −20^o^C, there was no obvious heat released observed and therefore no additional peaks were observed. This was due to the absence of any further unbound moisture or relatively low quantities of unbound moisture which could be below the minimum detection limits of the instrument [13].

Similarly, when the samples were thawed to room temperature at the same rate, heat absorption was observed to occur between 0^o^C to 15^o^C. The amount of energy absorbed during thawing was relatively larger than the energy released during the freezing stage. Similar behaviour was observed by Ref. [13] with sewage sludge. This increase in the absorbed energy was attributed to the simultaneous melting of moisture and possibly other frozen organic compounds. The depression of the freezing point was thus measured through the calculation of the enthalpy of heat released and absorbed during the thaw-freezing process using the trapezoidal rule. This was calculated to be 158 J/g for VIP, 162.3 J/g for UDDT, 123.55 J/g for ST-BW and 132.60 J/g for ST-wGW. The bound moisture was obtained subsequently from the total moisture content and the measured unbound moisture content from equation (8). The bound moisture for VIP sludge was found to be 52.77 %, for UDDT sludge at 51.48 %, ST-BW at 63.08 % and for ST-wGW at 60.37 %.

### 3.6 Statistical analysis

Tests for normality by Shapiro-Wilk tests and tests for homogeneity of variance by Levene's test was conducted which are pre-requisite for ANOVA. The Shapira-Wilk test for normality resulted with a p-value of 0.349, indicating the samples are from Normal distribution. For the Levene's test for homogeneity of variance, a p-value of 0.9955 indicated the homogeneity of residuals. The two-way ANOVA test indicated there is a difference in means between the methods and sample types.

To identify which of the means of the pair-wise combinations of methods and sample types are statistically significant, the Tukey's HSD posthoc tests were conducted. A multiple level comparison was carried out in two stages: first level between methods, and second level between methods for different sample types. The first level posthoc test between methods resulted in statistically significant difference between the groups WA - DSC and WA - TGA. However, the results between DSC and TGA was not statistically different. In the second level posthoc test, which is method per sample types of comparison, the difference of all method per sample types was not statistically significant except the following 4 interactions.

1. DSC:UDDT – DSC:ST-wGW
2. DSC:VIP – DSC:ST-wGW
3. TGA:VIP – TGA:ST-wGW
4. WA:UDDT – DSC:UDDT

Similarly, the multi-level Tukey posthoc comparison was carried out with sample types as the four independent groups at level one and comparison of sample type per method as level two. The results were the same 4 interactions as above (see Table 3).

## 4 Overall inferences – unbound-bound moisture interface

**Fig. 7:**
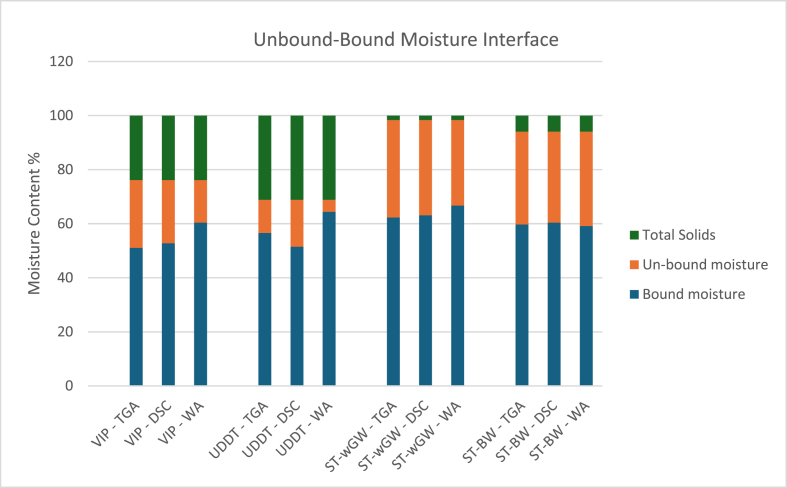
Unbound-bound moisture interface of FS samples from different experiments.

The absolute unbound moisture was different for different FS, since the initial moisture content varied between 99 % for the septic tank (with grey water) sludge to 68 % for the UDDT sludge. Thus, the unbound moisture fraction ranged from a low of 11.51 % for UDDT sludge to a high of 35.86 % for ST-wGW sludge. However, the unbound-bound moisture interface was primarily dependent on the bound moisture fraction which was consistently between 51.07 and 60.35 % for VIP sludge, 51.48–64.38 % for UDDT sludge, 62.29–66.71 % for ST-wGW sludge and 59.11–60.37 % for ST-GW sludge (Table 4). The proportion of the bound moisture fraction was the highest in the WA measurements. The results of both the DSC and TGA were relatively like each other for all samples (in Fig. 7).

**Table 3:** Multi-factor ANOVA analysis.

Source	Sum of squares	Degree of freedom	Mean square	F-ratio	p-value	Statistical significance
Main Effects						
Methods	240.7	2	120.33	11.463	0.00032	Yes
Sample types	417.6	3	139.21	13.261	0.0000262	Yes
Interactions						
Meth: Samp types	194.9	6	32.48	3.0094	0.02176	Yes

**Table 4:** Range of unbound-bound moisture interface for FS sludges.

	VIP	UDDT	ST-wGW	ST-BW
Unbound-bound moisture interface range %	51.07–60.35	51.48–64.38	62.29–66.71	59.11–60.37

From the statistical analysis using multi-factor ANOVA, there is a significant difference in the means between both the methods and the sample types. The posthoc test with multiple level comparison showed statistically significant different with results of WA with both DSC and TGA. However, the results between TGA and DSC were not statistically different. The methods per sample comparison yielded no statistically significant difference except with four interactions involving UDDT, VIP and ST-wGW samples.

It can thus be argued that the unbound-bound moisture interface fall in a range, instead of an operationally defined single value. This was because of the high variability of FS as reported in many other studies on FS, and the method used for determining. The possibility of removal of some of the bound moisture, especially the loosely bound interstitial moisture was another factor. The different proportions of bound moisture can also be attributed to the influence of interstitial moisture, besides the inherent limitations of the instrument and the methodology.

The BST experiment provided insights for the optimal design of networked solids' settleability for gravity based solid-liquid separation systems such as sludge drying beds, gravity thickeners, etc. However, BST was valid for lower solid's concentration since at higher solid's concentration, the solid's settling was slowed by the density difference between the solid particles and the liquid, thus requiring longer time to attain equilibrium [25]. This was evident with the results where the gel point values were at half the initial TS for samples with higher initial TS (VIP, UDDT and ST-BW) and was almost the same for ST-wGW sample. The presence of finer particles could be attributed for the colloidal suspension resulting in slow settling, evident from the presence of three phases in the BST.

While BST provided the extent of gravitational settleability, centrifugation experiments defined the extent of mechanical dewaterability with the application of external force up to nearly 2000 times gravity. The solid-liquid separation plateaued after the initial fall in the moisture content in the samples, both with respect to time as well as increased g-force. Further, with the polydispersity (presence of particles of various sizes) of the solid particles exhibited by all samples, the finer particles settled slower than the coarser particles and made it difficult to accurately identify the solid-liquid interface. This was like the presence of the colloidal suspension in the BST. The moisture content of the samples post centrifugation. did almost attain the unbound-bound moisture interface but the application of high centrifugal force and the longer exposure time make the test tedious. Additionally, the small difference in the final moisture content of the samples to its unbound-bound moisture interface could also be attributed to the poly-dispersity characteristics of the solid particles. Consequently, this test can be employed to investigate and predict the limits of mechanical dewaterability of sludges for different extents of digestion, thickening and conditioning, and to predict possible trends in solids’ handling equipment such as centrifuges, filter presses and plate presses, and not to determine the unbound-bound moisture interface. This concur with the attempted findings of the moisture content distribution carried out on sewage sludges [55].

The TGA tests provided valuable insights to the unbound-bound moisture interface as a drying test, with the drying rate, after an initial ramp up, starts to fall, indicating the falling drying rate period. As the most common technique to explore the kinetics of sample in drying, the TGA provided precise measurements of mass over time and temperature. Furthermore, non-isothermal drying of sample has assumed considerable importance as it minimizes the effect of moisture on the sample [56]. There has been substantial discussion regarding whether all the unbound moisture is removed at this point of change in the drying rate from increasing to first falling rate during non-isothermal drying, and whether some loosely bound interstitial moisture was also removed. There was consensus amongst the researchers that the major fraction of the moisture removed during the initial ramp-up period, in case of non-isothermal drying (similar to the constant drying rate period, in case of isothermal drying) is unbound moisture, and for practical purposes of designing, the start of the first falling rate period was considered as the beginning of removal of bound moisture, It was evident that the moisture required additional energy to overcome its binding energy. However, the quantity of sample used for the experiment was small, and consequently, the presence of any impurities since as small stones, sand, etc. could potentially influence the outcome, rendering the replicability of the results not always precise.

The DSC demonstrated highest reliability in terms of the process for determining the bound moisture content. The DSC thermograms provided clear peaks during the thaw-freezing experiment, and the determination of unbound moisture from the heat enthalpy was simple. However, DSC required the presence of at least some quantity of unbound moisture which could be a major limiting factor for the application of DSC. While for all practical purposes, the raw FS from the different OSS do have some quantity of unbound moisture. Also, similar to the TGA, the small quantity of the sample can affect its replicability.

The moisture sorption isotherm of FS from the WA meter shows constant measurement of water activity close of 1 and a gradual decrease starts when the sludge moisture is removed beyond a certain point. WA meter measured the relative humidity in the headspace independent of the sample in the vapour phase, thus allowed for an accurate determination of the water activity. The WA measurements were the quickest, simplest and cheapest amongst the methods applied [14]. also found the sorption isotherms to be the most accurate method to determine the characterization of bound moisture, and literature has shown that any alteration to the moisture boundness in terms of conditioning and mechanical dewatering do not alter the bound moisture repartition, making WA robust.

## 5 Conclusions

The unbound-bound moisture interface lie within a range for all FS samples, between 51.07 and 60.35 % for VIP sludge, 51.48–64.38 % for UDDT sludge, 62.29–66.71 % for ST-wGW sludge and 59.11–60.37 % for ST-GW sludge. The bound moisture fraction does not completely dependent on the initial moisture content, it does influence the fraction to an extent, as observed with the ST-wGW sample. Despite the heterogeneity nature of the samples, the TGA, DSC and WA experiments provided with reasonable accuracy, the bound moisture fraction with the centrifugation results close to the unbound-bound moisture interface, especially with the high TS samples. Although all the methods have their advantages and disadvantages, the WA measurements outperform the TGA and DSC experiments due to its accuracy, replicability, low influence of the variability of the sample, ease of conducting the experiment, rapidity and repeatability. The multi-variate analysis using multi-factor ANOVA indicated statistically significant differences between the methods and sample types, but no significant difference in the interactions between methods and sample types.

## CRediT authorship contribution statement

**Arun Kumar Rayavellore Suryakumar:** Writing – original draft, Visualization, Validation, Methodology, Investigation, Formal analysis, Data curation, Conceptualization. **Edwina Mercer:** Writing – review & editing, Supervision. **Jonathan Pocock:** Writing – review & editing, Supervision, Methodology, Conceptualization. **Santiago Septien:** Writing – review & editing, Visualization, Supervision, Methodology, Investigation, Conceptualization.

## Declaration of competing interest

The authors declare that they have no known competing financial interests or personal relationships that could have appeared to influence the work reported in this paper.
